# Mitigating Measurement Inaccuracies in Digital Twins of Construction Machinery through Multi-Objective Optimization

**DOI:** 10.3390/s24113347

**Published:** 2024-05-23

**Authors:** Misganaw Abebe, Yonggeun Cho, Seung Chul Han, Bonyong Koo

**Affiliations:** 1 Department of Mechanical Engineering, Kunsan National University, Gunsan 54150, Republic of Korea; misge98@gmail.com; 2 Korea Construction Equipment Technology Institute, 52, Saemangeumsandan 2-ro, Gunsan 54002, Republic of Korea; choyg1205@koceti.re.kr

**Keywords:** excavator finite element model, surrogate model, multi-objective optimization, Gaussian process regression, TOPSIS, digital twin

## Abstract

The advent of digital twins facilitates the generation of high-fidelity replicas of actual systems or assets, thereby enhancing the design’s performance and feasibility. When developing digital twins, precise measurement data is essential to ensure alignment between the actual and digital models. However, inherent uncertainties in sensors and models lead to disparities between observed and predicted (simulated) behaviors. To mitigate these uncertainties, this study originally proposes a multi-objective optimization strategy utilizing a Gaussian process regression surrogate model, which integrates various uncertain parameters, such as load angle, bucket cylinder stroke, arm cylinder stroke, and boom cylinder stroke. This optimization employs a genetic algorithm to indicate the Pareto frontiers regarding the pressure exerted on the boom, arm, and bucket cylinders. Subsequently, TOPSIS is applied to ascertain the optimal candidate among the identified Pareto optima. The findings reveal a substantial congruence between the experimental and numerical outcomes of the devised virtual model, in conjunction with the TOPSIS-derived optimal parameter configuration.

## 1. Introduction

An excavator is a highly automated soil/rock excavating loading machine that can work effectively and conveniently in challenging situations and on constrained routes [1,2]. This enables it to meet numerous engineering specifications and find widespread application in the development of infrastructure, mining, agriculture, transportation, and other fields [3]. Excavator parts must function consistently under unpredictable operating conditions and are exposed to high loads because of extreme working conditions. Excessive stress levels might damage essential excavator components like the boom, arm, and bucket, which will have a negative impact on the machine’s output. Thus, it is crucial to deliver equipment with the highest level of reliability, while maintaining the lowest possible weight and cost and satisfying all loading requirements for a safe design. Currently, weight is a key consideration in the design of machine parts, since performance is directly correlated with the power-to-weight ratio [4,5]. It will also assist in reducing the excavator’s overall cost. Thus, optimization is necessary to reduce the boom’s weight and to improve the machine’s performance. Currently, physical assets can be digitally replicated and analyzed using digital twin technology [6,7,8]. This enables the performance analysis and optimization, scenario simulation, and outcome estimation of the intended model. This means designers should generate a high-fidelity FEA model for the excavator’s digital twin to test its mechanical stability under a variety of loading scenarios. When designing excavator parts, force and strength analyses are likewise crucial factors [9,10]. Thus, to determine the pressure load at each cylinder, the forces at each joint, and the distribution of stress at the boom and arm, this study developed an FEA model with a multibody dynamic analysis in COMSOL.

However, before considering the use of the developed FEA model simulation to enhance test design or evaluation, it is necessary to comprehend the degree to which a simulation model approximates the actual system and the domains in which it is more restricted with respect to the intended use. Several activities are described in the previous studies [11,12] regarding model validation; these activities can be broadly categorized as external validation and sensitivity analysis. The process of comparing the output of a model with observations from the system that is being modeled is an example of external validation. External validation is enhanced by conducting an uncertainty analysis of a model, which examines the range of model output that arises from normal variations in model inputs. The uncertainty analysis can be used as a benchmark to determine if variations between the system’s observed behavior and the model’s predicted behavior can be accounted for by model uncertainty. Thus, this study proposed a multi-objective optimization, along with surrogate modeling, to achieve computational effectiveness in determining the best configuration of the excavator’s uncertain parameters while verifying the FEA model with experimental results.

In the early stages of the design process, surrogate models replace the expensive FEA approaches and reduce computational time significantly. The basic idea of using a surrogate model is not wholly novel. Several researchers have studied and applied a variety of surrogate models, such as the polynomial model [13], the radial basis function [14], polynomial chaos expansion [15], the Kriging method [16], and the adaptive Gaussian process [17]. In addition, generating the sample data to develop a surrogate model is a critical activity, since surrogate models are built using the training samples in the parameter space. For this reason, other studies have considered surrogate modeling, such as Sobol sequence sampling [18], uniform sampling [19], Latin hypercube sampling [20], and central composite sampling [21], for updating FE models from the point of view of the design of experiments. In general, there is no definitive standard for determining which surrogate model is superior, and none of them can handle all engineering problem scenarios [22]. Various surrogate models frequently result in different design outcomes and accuracy. Despite the increasing recognition of surrogate models’ exceptional performance, as the authors are aware, using a surrogate model is still relatively new in regards to construction equipment, particularly in the optimization of the hydraulic excavator’s working device.

This study investigated the controllable excavator’s uncertain parameters, which may have influenced the output difference between the actual (experimental) test result with the developed multibody dynamic and FEA model. The contribution of this study can be summarized as follows: (1) using the COMSOL Multiphysics software, a three-dimensional parametric FEA method and multibody dynamic model of the excavator is provided. The modeling, assembly, and analysis of the excavator FEA model can be controlled parametrically, and the conditions can be executed in an easy and accurate manner, according to the actual operating conditions; (2) with the combination of the finite element analysis and stress experiment tests, the stress at the boom and arm and the pressure load at the cylinders of the excavator is effectively studied; (3) to reduce the relative error between the experimental and FEA results, this study also proposed a multi-objective optimization, along with surrogate modeling, considering four influential (uncertain) parameters, such as the external load angle at the bucket tip, the arm cylinder stroke, the boom cylinder stroke, and the bucket cylinder stroke. To reduce the computation time, the high-fidelity FEA and multi-body dynamic models were first replaced by a Gaussian process regression surrogate model. Then, to find the best candidate for the uncertain parameters, multi-objective optimization using a genetic algorithm in MATLAB R2023b was used to find the Pareto front for pressure load at the bucket, arm, and boom cylinder. To find the best combination of parameters from the Pareto front, Technique for Order of Preference by Similarity to Ideal Solution (TOPSIS) was finally employed. The TOPSIS solution with the lowest relative error was quantitatively validated through comparison with the outcomes of an experiment, and it was numerically verified through comparison with the results of a numerical simulation.

The study’s remaining sections are arranged as follows: Section 2 discusses model development, including the 3D model development of the excavator, FEA, the multibody dynamic model, the experimental setup, and the formulation of the Gaussian process regression model, with the verification methods. Section 3 presents the results and a discussion, including the formulation of the multi-objective model. Finally, the conclusion is presented in Section 4.

## 2. Materials and Methods

### 2.1. Excavator’s 3D Model Development

**Figure 1:**
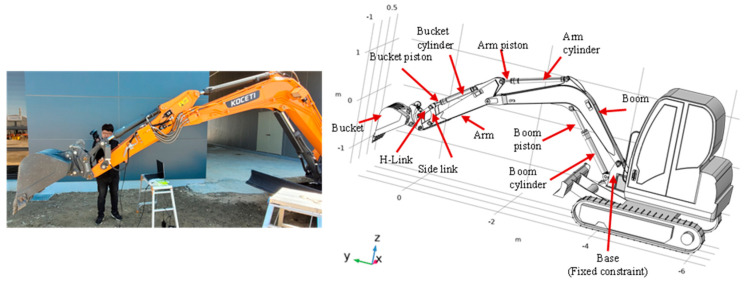
3D model of the mini excavator (DX55 MT−5K).

The operating mechanism of a full hydraulic mini excavator includes a bucket, arm, boom, bucket cylinder, arm cylinder, boom cylinder, connecting pin shaft, and other components. In order to guarantee the reliability and accuracy of the working mechanism under a variety of conditions, a three-dimensional model of the working mechanism of a full hydraulic excavator was developed. As shown in Figure 1, a one-to-one 3D model of the DX55-5K/DX55MT-5 mini-excavator was developed in CAD software after conducting a 3D scan. The following are the fundamental geometric structure parameters of the excavator: The boom measures 2.82 m in length, the arm is 2.14 m in length, the maximum disposal height is 3.90 m, the maximum excavation depth is 3.96 m, and the maximum excavation radius is 6.3 m. More detailed information about the geometry and structure of the excavator can be found in the manufacturer’s catalog [23]. COMSOL Multiphysics software was then used to perform the FEA and multibody dynamic model.

### 2.2. Finite Element Model

**Figure 2:**
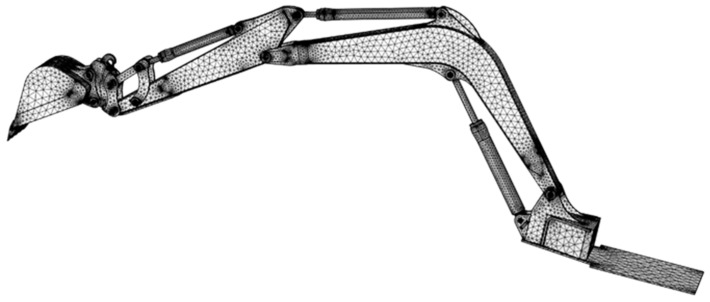
Finite element model of the excavator.

Following the development of the 3D model, we developed a finite element model for analysis purposes, as depicted in Figure 2. Some of the procedures involved in the process of developing a finite element analysis include meshing, applying boundary conditions, assigning material properties, and other similar steps. The .igs file was imported into the COMSOL Multiphysics software. After importing the excavator’s CAD data, we generated and meshed the surfaces. To reduce the computational time, the crawler track walking device was excluded from the FEA model, and as shown in the figure, the main components such as the boom, arm, links, and bucket are included. The COMSOL program is utilized to carry out the modeling and analysis of mechanical characteristics.

In the FEA model, due to the complexity of the geometry, the study used a tetrahedral-free mesh. First, the individual components of an excavator are meshed independently, and then they are joined by providing the appropriate connectivity between the components. In total, 78,691 and 148,612 mesh elements for the arm and boom components, respectively, are used. In addition, the material for steel plates used for excavator manufacturing is SWS490A. The material Young’s modulus is 210 GPa; the Poisson’s ratio is 0.3; the yield strength is 315 MPa, the density is 7.8 × 10^3^ kg/m^3^, and the gravitational acceleration is 9.8 m/s^2^. Table 1 also provides a summary of the material properties. An elastic-plastic material model with a bilinear isotropic hardening relationship is also used in this analysis.

### 2.3. Multibody Dynamic Model

Multibody dynamics is the study of the dynamic behaviors of mechanical systems composed of rigid and/or flexible bodies connected by joints. The bodies undergo translational and rotational motions caused by applied forces, torques, and constraints. The multibody dynamic model was also developed in COMSOL Multiphysics software. COMSOL is a software package that uses finite element modeling to simulate several physical phenomena. It also includes a module for analyzing the dynamics of multiple bodies. This module enables the simulation of the dynamic characteristics of rigid or flexible objects, or a combination of both, that are linked together by joints that permit and restrict specific degrees of freedom. The system of partial differential equations (PDEs) is formulated using the Lagrangian approach and then solved for the Dirichlet and Neumann boundary conditions that are imposed on the domains. Both rigid and flexible bodies can be subjected to forward and inverse dynamics. In the case of flexible bodies, the model can generate the material’s elastic displacement field, which can then be used to calculate stresses and strains.

As mentioned above, the assemblies of rigid components, flexible components, or a combination of both are modeled using the COMSOL multibody dynamics interface [24]. It is possible to define flexible components using solid, shell, or beam elements. We can also use this physics interface to perform a system analysis, as well as a detailed component analysis of a mechanical system simultaneously. The features that are employed to model the connections are joints, attachments, rigid domains, springs, and dampers. For modeling various kinds of connections between components, COMSOL offers a variety of joints. Joints use attachments to create a connection, primarily between flexible parts but also with rigid bodies. A rigid domain directly connects one rigid component to another rigid or flexible component by modeling the rigid component.

In our case, the arm and the boom are considered as flexible components, while the other components are rigid bodies, so we used an attachment to connect all the components, since the attachment connection works with both flexible and rigid bodies. Regarding the joints, since the piston/rod and the cylinder have one translational degree of freedom between the two connections, the prismatic joint [25] was used to create translational motion for the bucket, boom, and arm pistons/rods in their respective cylinders. A hinge joint was used for the other joints, such as a boom with an arm cylinder, a base with a boom, a boom with an arm, etc., because the joints on this connection must be free to rotate relative to each other at the joint’s axis.

#### 2.3.1. Operating Cycle

**Figure 3:**
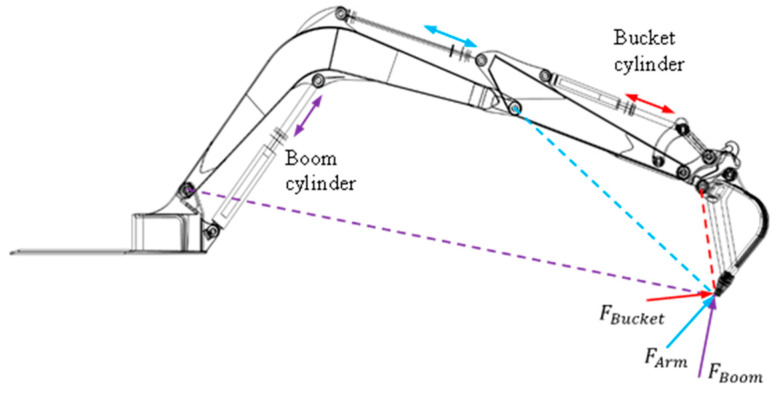
Excavator’s free body diagram.

The working cycle of an excavator consists of digging, swinging, dumping, and swinging back to the initial position. As shown in Figure 3, the excavator is operated by controlling three cylinder lengths: the bucket cylinder, which controls the angle of the bucket; the arm cylinder, which controls the angle between the outer boom and the inner boom; and the boom cylinders, which raise the boom (bucket). In this study, we used the piston’s extension to specify an operating cycle, but it is also possible to control the motion in an angular way.

#### 2.3.2. Constraint and Loading

Due to the large weight of the excavator’s component, the gravity force is incorporated into the FEA model. The maximum working load of this mini excavator is also considered to be 41 kN, which was taken from the DX55MT−5K mini-excavator brochure [23]. In this study, the external load is applied to the bucket tooth, and its loading mode is determined according to the stress test experiment. The displacement degree of freedom of the base component is a fixed constraint.

### 2.4. Experimental Setup

**Figure 4:**
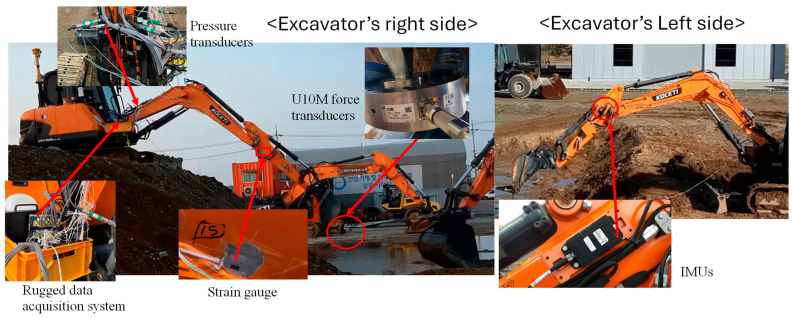
Experimental testing instruments.

To validate the proposed multibody dynamic and FEA model of the excavator, a stress test experiment was carried out in the Korea Construction Equipment Technology Institute (KOCETI), under a typical working conditions, based on the structure stress test criteria of the mini-excavator (DX55 MT-5K). The primary measuring component of the strain measurement test is the strain gauge. It is a conventional non-destructive technique that is frequently used in the engineering machinery sector [26]. Figure 4 depicts the test setup of the experiment, which was carried out outdoors. Moreover, the main test instruments are also shown in Figure 4, and their functions are introduced in the following section.

#### 2.4.1. Instruments for Stress and Pressure Load Test

**Figure 5:**
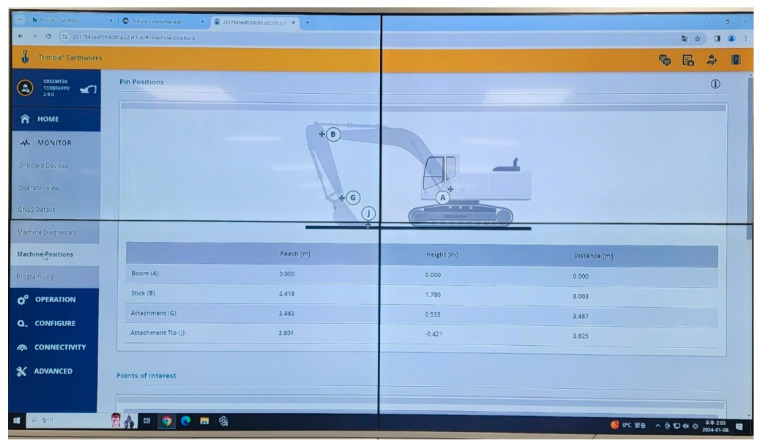
IMUs tracking locations.

The instruments for stress and pressure load test experiments are demonstrated, as shown in Figure 4. The main instruments include: (i) KFGS general-purpose foil strain gauges (KFGS-5-350-C1-11 L5M3R). These instruments are used to achieve the strain value. By using general-purpose foil strain gauges, strain in the uniaxial direction can be obtained. Based on these strain results, the stress at the test point can be easily determined. (ii) Inertial measurement units (IMUs) were used to acquire the position of the boom cylinder, stick cylinder, and bucket cylinder, which determine the excavator’s spatial attitude and working conditions. As shown in Figure 5, we track the location of the joint between the arm and boom (point B), the joint between the arm and bucket (point G), and the tip of the bucket (point J) from the origin of the joint between the boom and the base component (point A). (iii) An HBK U10M force transducer was used to determine the magnitude of the loads that are applied on the bucket tooth. (iv) A pressure transducer (HySense PR 400) was used to measure the pressure load at each cylinder. The sensor was applied to both the pulling and pushing sides of the cylinder to measure the pressure load on both sides, and then it was connected to SomtXR DAQ for collecting and recording the data from the cylinder on a computer. The SomatXR is a modular, robust data-gathering device that can be used for any type of measurement [27]. Using the robust SomatXR data collection technology in challenging conditions makes data collection easier. The modules can be combined flexibly via the Ethernet interface to suit a variety of applications, and they can be operated in a wide temperature range.

#### 2.4.2. Process of Testing and Excavator’s Test Position

**Figure 6:**
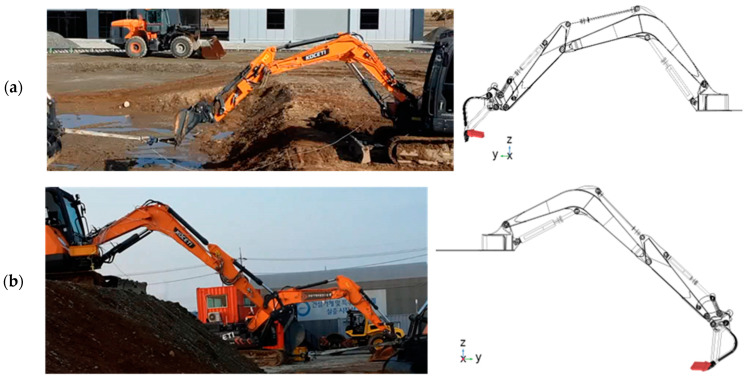

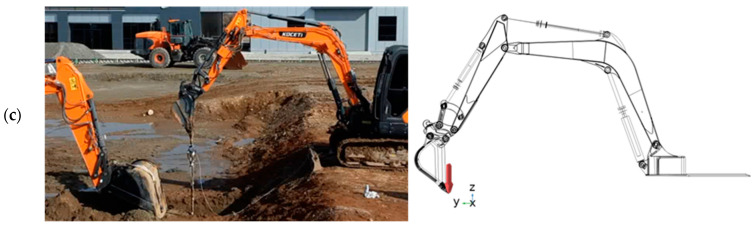
Experimental setups with the corresponding simulation setups: (**a**) Case-1; (**b**) Case-2; (**c**) Case-3.

This study conducts stress test experiments under typical excavator working conditions, adhering to standards and regulations. The representative working condition of the excavator status, which is selected for the test experiment, and the corresponding simulation model setups are shown in Figure 6. The three cases, which were set in different working positions, are shown in Figure 6. In addition, we used different load conditions, i.e., for Case-1, only the arm cylinder was active; for Case-2, the bucket cylinder was active; and for Case-3, the boom cylinder was active in an upward direction. The final working position (location coordinate points) is shown in Table 2.

### 2.5. Theory on Surrogate Model Development

As described in the introduction section, surrogate models are utilized as fitness functions in evolutionary algorithms. The fundamental idea behind this approach is to use an approximate model in place of a computationally expensive model [28]. Surrogate models are developed in one of three distinct ways [29]: data-fit methods, simplified models, and projection-based approaches, i.e., simplified models include algorithms for spatial dimensionality reduction, which are constructed by simplifying the simulated system [30,31].

The data-fit methods establish a mapping between input and output latent functions. Gaussian processes [32], support vector machines (SVM) [33], and neural networks (NN) [34] are the most common approaches to developing a surrogate model. This study employed Gaussian process regression as a surrogate model to reduce the computationally time-consuming finite element model. We also used GPR to demonstrate the feasibility of estimating the propagation dynamics of responses with different sets of input parameters.

#### 2.5.1. GPR Model Formulation

GPR is a class of supervised machine learning algorithms for which it is sufficient to use a few parameters to make a prediction. A Gaussian process is considered as an infinite extension of the multivariate normal distribution. The relationship between the input vector and the output parameter can be written as:

(1) $$y_{i}=f\left(x_{i}\right)+\varepsilon ,$$

where $f\left(x_{i}\right)$ is the function representing the independent variable for $i^{th}$ observation, and ε representing the noise added to the observed variables. In our study, the extracted bucket cylinder stroke (BuCS), arm cylinder stroke (ACS), boom cylinder stroke (BoCS), and load angle (F_angle) at the tip of the bucket are considered as independent variables. The target variables considered as dependent variables are the pressure load at the bucket, arm, and boom cylinder, assuming a zero-mean value, $\varepsilon \sim N(0,\sigma_{n}^{2})$, where $\sigma_{n}$ is the standard deviation of noise. The prior distribution of the training sample is obtained as $y=N(0,K+\sigma_{n}^{2}I)$, where I is the $n^{th}$ order unit matrix.

Given a training dataset $D=\left\{X,y\right\}$, the best estimate of the dependent variable $y^{*}$ of a new test dataset is found. GPR is a non-parametric regression model that constructs a relationship between X and y based on the geometric positions of $x_{i}$ for all i within the feature space. y is a collection of samples from an n-variate Gaussian distribution, and hence, GPR provides the expected value and the variance of $y^{*}$. A Gaussian function is completely specified by its mean function $m_{x}$ and a covariance function $k\left(x,x^{\prime }\right),$ which is also known as the kernel. For all, $x^{\prime }$, $m_{x}=E\left(f\left(x\right)\right),$ and $k(x,x^{\prime })=Cov(f(x),f(x^{\prime }))$. In the present work, a zero mean is assumed. A squared exponential kernel is used for all cases.

For the training dataset $D=\left\{X,y\right\}$ and the testing input vector $X^{*}$, the joint distribution of the observed dependent variable y and unobserved variable $y^{*}$ can be written in a matrix form as:

(2) $$\left(\begin{array}{c} y\\ y^{*} \end{array}\right)\sim N\left(\begin{array}{c} 0\\ 0 \end{array}\right),\left[\begin{array}{cc} K_{XX} & K_{X^{*}X}\\ K_{XX^{*}} & K_{X^{*}X^{*}} \end{array}\right]$$

For a set of inputs $X\in R^{N\times D}$ and corresponding outputs $y\in R^{n}$, the outputs $y^{*}$ can be estimated from the new sets of inputs $X^{*}$ by modeling the function y as a GP, i.e., if the observed data is y and the unobserved data is $y^{*}$ coming from a GP, concatenating y and $y^{*}$ results in a multivariate normal distribution, with the mean and covariance structure given by Equation (3) [32,35]. Now, as y is observed, $y^{*}$ can be modeled using the conditional distribution of a multivariate normal, given by:

(3) $$p\left({y^{*}|X}^{*},X,y\right)=N\left(K_{X^{*}X}K_{XX}^{-1}\right)y,K_{X^{*}X^{*}}-K_{X^{*}X}K_{XX}^{-1}K_{XX^{*}}$$

In this study, $y^{*}$ represents the pressure load at the bucket, arm, and boom cylinder.

#### 2.5.2. Verification of the Model

To ensure that the approximation model is sufficiently accurate, we evaluate numerical metrics using a root mean square error RMSE. The RMSE is calculated as the square root of the residual variance of the individual differences. It indicates the degree of correspondence between the estimated values and the actual values of the data. RMSE is generally an absolute metric for determining the suitability of a model. When RMSE is smaller, it indicates a more accurate fit. When building a prediction model, RMSE serves as a suitable and accurate statistic to illustrate the expected responses. The RMSE equation is given as:

(4) $$RMSE=\sqrt{\frac{1}{n}\sum_{i=1}^{n}{\left({\hat{y}}_{i}\left(x\right)-y_{i}\right)}^{2}}$$

## 3. Results and Discussion

### 3.1. Convergence Study of FEM

**Figure 7:**
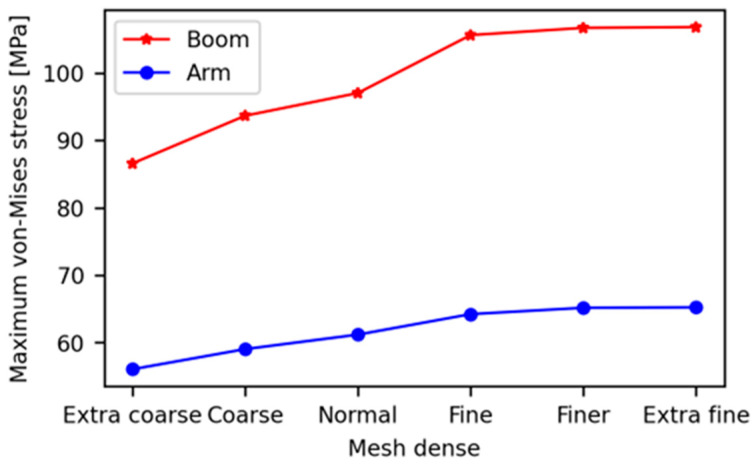
Convergence study of the FEM.

Firstly, we conducted a convergence study to demonstrate the accuracy (adequacy) of the developed FEA model. To find the appropriate mesh element size parameter configuration for the flexible component (boom and arm), we extracted the parameter setting in COMSOL, based on the specified size (from extra coarse to extra fine mesh size), as indicated in Table 3. Under the same boundary constraints and loading conditions as Case-1, the maximum von Mises stresses of the boom and arm components of the excavator were analyzed. The numerical simulation results of the maximum von Mises stress at each parameter setting is shown in Figure 7. As the result shows, the maximum von Mises stress values on both the boom and arm components do not show a significant change after the fine mesh size configuration, so this study used a fine mesh for the two flexible components.

### 3.2. Numerical Results Analysis

**Figure 8:**
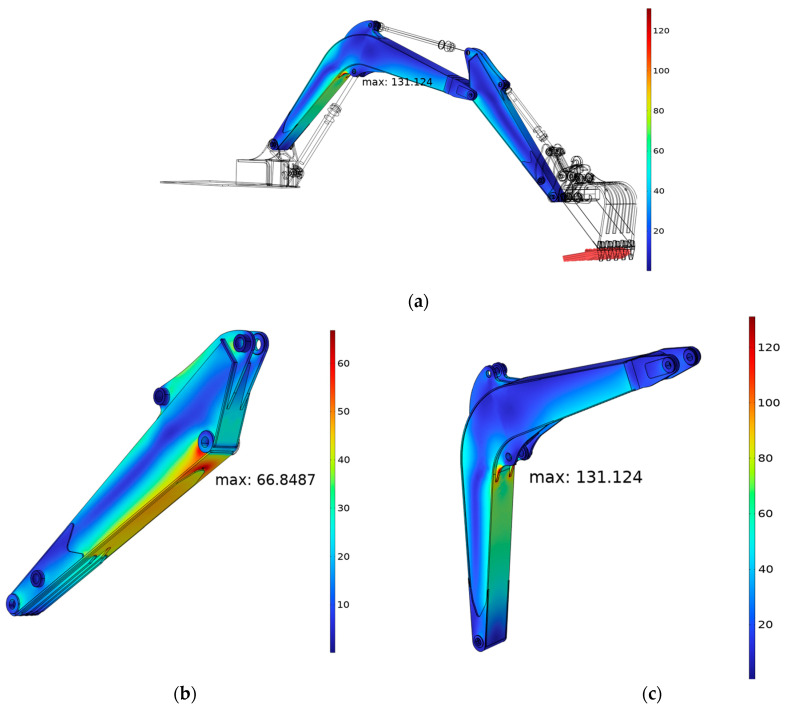
Contour plots of von Mises equivalent stress of the excavator: (**a**) excavator; (**b**) arm; (**c**) boom.

The distributions of the von Mises stress of the flexible components (arm and boom) for the Case-1 shown in Figure 6 are shown in Figure 8. In this case, the external force was 40.6 kN, the same as in the experimental case, and as can be seen from the von Mises stress contour plot, the maximum von Mises stress on the arm component is 66.85 MPa and 131.12 MPa on boom, which are less than the yield stress. The contour of the boom component also shows a high stress concentrated on the boom piston supporter plate and its welding area, which is an acceptable result. To validate the FEA model, a field test was conducted. In addition, the following section details the inquiry to establish the optimal uncertain operation settings for correlating the simulated and actual test results.

### 3.3. Surrogate Model Development

#### 3.3.1. Design of Experiment (Sampling Strategy)

The investigated parameters of this study are external force angle at the bucket tip, the arm cylinder stroke, the boom cylinder stroke, and the bucket cylinder stroke for the three working conditions (cases). As discussed in Section 2.4, IMUs were used to measure the excavator’s bucket, arm, and boom movements, but for ease of control in the simulation, the study used the cylinder stroke (displacment) to obtain the measurements. In addition, the external force angle was derived based on geometric relationships. As a result, the uncertainty of these controllable input parameters may affect the pressure load value induced at the cylinders; therefore, this study explored the uncertainty effect of these four input parameters to reduce the simuation and the actual pressure load values. The mean values are set to the same value as those in the testing. When we experimented, we used IMUs to track the respective joint location while setting the working position, but in the simulation model, we controlled the cylinders’ stroke using the piston/rod displacement.

Johnson et al. [36] presented space-filling Latin hypercube sampling as one of the sampling methods for the designing of experiments. In this investigation, the free-access MATLAB code of the Latin hypercube algorithm was utilized to acquire 40 training examples. We then generated an additional 40 random samples to verify the developed surrogate model, resulting in a total of 80 simulations to determine the pressure load at each cylinder.

#### 3.3.2. Sensitivity Analysis

**Figure 9:**
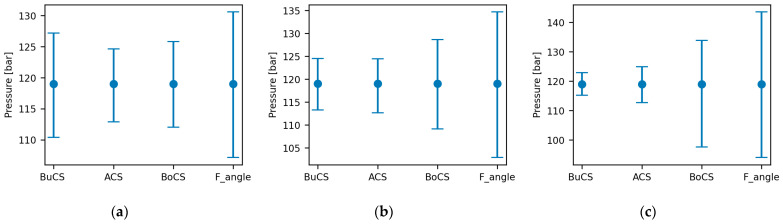
Effects of the uncertain parameters on the model output of: (**a**) pressure at the bucket cylinder; (**b**) pressure at the arm cylinder; (**c**) pressure at the boom cylinder.

Before proceeding to develop the surrogate model with the selected uncertain parameters, the response of the three-model output was investigated through a one-at-a-time parameter sensitivity analysis [37,38]. The investigation involves analyzing the sensitivity of each uncertain parameter, while keeping the other parameters as constant values. The values of these constants are the mean values that are presented in Table 4. As demonstrated in Figure 9, all parameters have a considerable effect on all responses, particularly the force angle. Thus, all parameters are employed in this study in search of an optimal parameter configuration for reducing the relative error between the experimental and simulation results.

#### 3.3.3. Surrogate Model Verification

**Figure 10:**
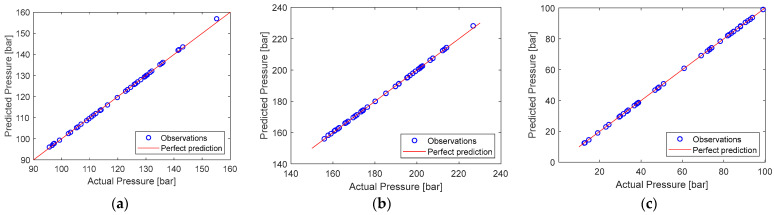
Predicted vs. Actual pressure for Case-1: (**a**) at bucket cylinder; (**b**) at arm cylinder; (**c**) at boom cylinder.

Using the developed multibody dynamic models, we evaluated the pressure load at the bucket cylinder, arm cylinder, and boom cylinder for the generated Latin hypercube design samples. Then, we used the DPR surrogate model to approximate the functional relationships between the three response measures and the four uncertain parameters for each case. In addition to the GPR model, the study developed SVM [39], ANN [40], and third-degree polynomial regression models for comparison. Table 5 summarizes the model verification of all four surrogate models for all cases, which were tested using RMSE values on test sample data. As the results show, in all cases, the RMSE result of the proposed GPR model exhibited the highest accuracies when compared to the other models, confirming that the generated GPR model accurately predicts the observed data. Figure 10 also displays the predicted vs. actual pressure value of Case-1 for each model, which shows that the developed GPR model predicted the random testing data perfectly.

### 3.4. Multi-Objective Optimization

#### 3.4.1. Optimization Formulation

This study develops the multi-objective function based on the relative error percentage between the experimental and numerical simulation results, and presents it as follows:

(5) $$f\left(x_{exp},x_{sim}\right)=\left|\frac{x_{exp}-x_{sim}}{x_{exp}}\right|\times 100\%,$$

where $x_{exp}$ is the experimental test value, and $x_{sim}$ is the numerical simulation value.

Thus, the objective is to find the optimal uncertain parameter configuration with the minimum relative error between the experimental and the simulation data. The formulation of the objective function will be as follows:

$$\underset{X}{\min}{(f}_{BuP}\left(x\right),{(f}_{AP}\left(x\right){,(f}_{BoP}\left(x\right))\;$$

subject to 

(6) $${lb}_{i}\leq x_{i}\leq {ub}_{i}$$

where $x_{i}=(x_{1},x_{2},x_{3},x_{4})$ are the uncertain parameters (bucket stroke, arm stroke, boom stroke, and external load angle at the bucket tip), and $f_{BuP}$, $f_{AP}$, and $f_{BoP}$ are the objective function of pressure load at the bucket cylinder, arm cylinder, and boom cylinder, respectively.

#### 3.4.2. Result of Multi-Objective Optimization

For this investigation, the multi-objective genetic algorithm was utilized to obtain the Pareto front of the three objective functions. To regulate the elitism of the genetic algorithm, the Pareto fraction and the distance function are utilized. Both the Pareto fraction option and the distance function contribute to the preservation of diversity on a front by giving preference to candidates that are located relatively far away from the front. The Pareto fraction option restricts the number of individuals situated on the Pareto front. For this study, we employed a population size of 400, a Pareto percentage of 0.5 (which corresponds to 50% of the total population size), and 100 generations.

To determine which combination would be the most effective, several multiple-criteria decision-making methods have been proposed for different areas, including the analytical hierarchy process (AHP) [41], elimination and choice translating reality (ELECTRE) [42], the technique for order of preference by similarity to the ideal solution (TOPSIS) [43], multi-level linguistic decision-making methodology (multi-level LDM) [44], and the preference ranking organization method for enrichment evaluation (PROMETHEE) [45]. Various studies have also compared these methods from different perspectives [46,47]. In this specific investigation, the TOPSIS method is employed, which is a commonly used methodology in numerous fields, offering faster computational time compared to other methods.

**Figure 11:**
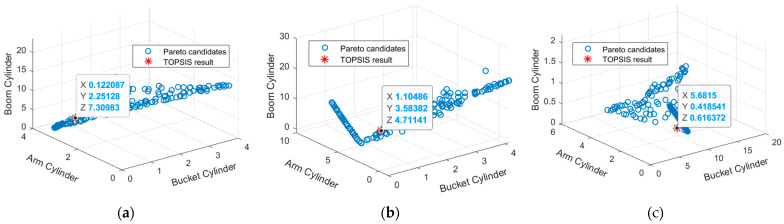
Pareto fronts for minimizing the relative error of the bucket, arm, and boom cylinder: (**a**) Case-1; (**b**) Case-2; (**c**) Case-3.

When selecting an option, TOPSIS is utilized to determine which alternative exhibits the shortest Euclidean distance to the positive ideal solution and the Euclidean distance that is the farthest from the negative ideal solution. Positive ideal solutions are those that exhibit the highest level for all of the attributes that are taken into consideration, whereas negative ideal solutions are those that show the lowest attribute values; for more information, refer to the book written by Tzeng and Huang [48]. The results of the Pareto front and TOPSIS tests for the same weights of the bucket, arm, and boom cylinder pressure output for the three cases are shown in Figure 11.

A finite element simulation was performed using the obtained optimal parameter configuration values described in Table 6. As shown in Table 7, the relative error percentage for all cases has improved. For example, in Case-1, the percentage of the relative error of the pressure load on the boom cylinder was reduced from 21.43% to 7.58%. In Case-2, the percentage of the relative error of pressure load on the boom cylinder reduced from 12.81% to 4.546%, and in Case-3, the percentage of the relative error of the pressure load on the bucket cylinder reduced from 38.60% to 5.586%, which demonstrated that the proposed multi-objective optimization based on the GPR surrogate model can help validate the developed FEA model.

**Figure 12:**
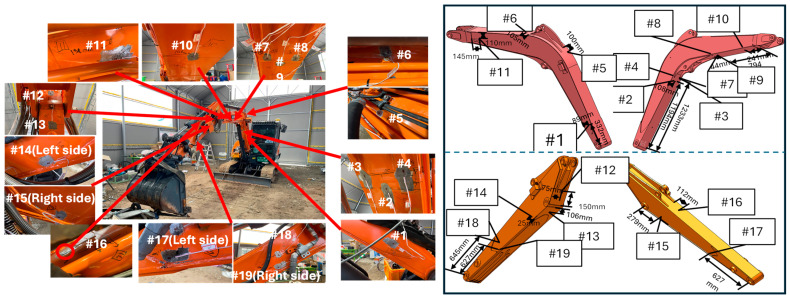
Strain gauge locations.

In addition, using the optimal configuration of the parameters, we validated the stress at different locations. The stress distributions of the test points are shown in Figure 12. A total of 18 test points located on the excavator boom and arm components are selected to achieve the stress results.

**Figure 13:**
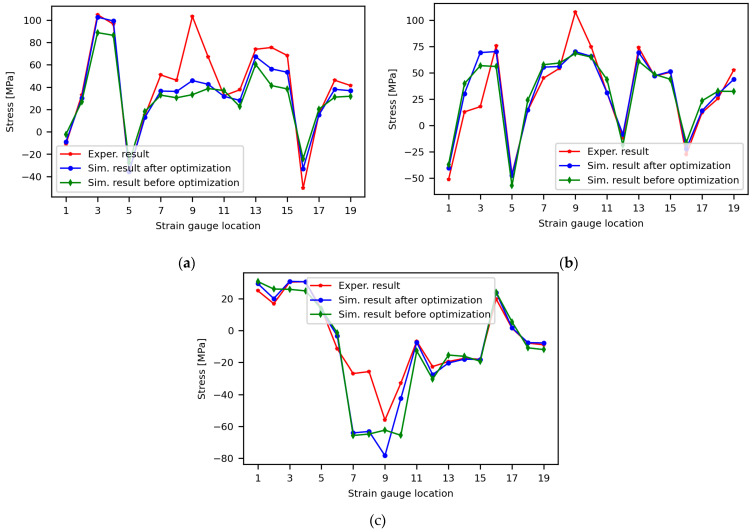
Stress comparisons: (**a**) Case-1; (**b**) Case-2; (**c**) Case-3.

Figure 13 shows that the stress value of the simulation result, before and after optimization, has been verified by the experimental result at the chosen location of the boom and the arm. As shown in Figure 13a, most of the experimental stress values appear to correspond with the simulation result using the optimal parameters for Case-1, except for location point 9, where the experimental result is significantly higher. The experimental result shows 103.18 MPa, and the simulation result is 45.85 MPa.

**Figure 14:**
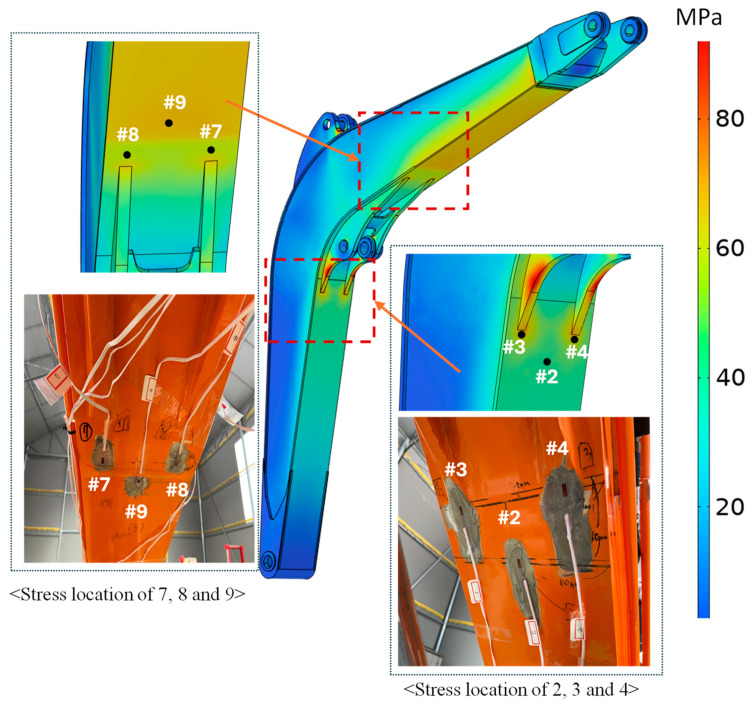
The distribution of the first principal stress of the boom component in Case-2.

Similar to Case-1, as shown in Figure 13b, while using the optimal parameters, most of the stress values match the experimental result for Case-2, except for in a few locations. For example, as was the result for Case-1, the stress value at Point 9 shows a significant difference from the experimental value. In addition, the experimental stress value at Point 3 is significantly lower than the simulation result. In the experiment, the stress value at Point 3 was 17.83 MPa, while the simulation result was 69.12 MPa. However, Figure 14 illustrates that the stress values at points 4 and 3 should not significantly differ, given their symmetry and the application of a uniform bending load to the bucket’s tip. The experimental and simulation results at Point 4 show 75.46 MPa and 70.14 MPa, respectively, which are almost the same as those obtained with the simulation at Point 3. Figure 13c also shows the validation result of the simulation’s stress result for Case-3. As the result shows, using the optimal parameters, the numerical simulation results match the experimental result, except at points 7, 8, and 9; however, the trends are the same. In general, based on this result, the proposed optimization model can show how the developed virtual model of the mini excavator matches the results of the actual test by avoiding the uncertainty effect.

## 4. Conclusions

This study developed a multi-body dynamic model of a mini excavator in COMSOL to determine the external loads acting on the boom, arm, and bucket cylinders, as well as to simulate the movement of the excavator’s bucket, arm, and boom. The proposed assembly method was utilized for developing the FEA model, while considering the spatial relationship between the local and global coordinate systems; the convergency test indicates that the developed FEA model is adequate. The mechanical behaviors of the excavator were subsequently predicted through the implementation of the developed FEA method at various operating conditions. To validate the developed FEA model, an actual stress test investigation was also conducted. In addition, a GPR surrogate model was developed in order to replace the computationally expensive FEA model and to identify an optimal uncertain parameter configuration that is capable of matching the results of the FEA model with the results of the experiments. In comparison to the SVM, ANN, and polynomial regression models, the verification accuracy results from the GPR surrogate model demonstrated that the proposed model has a significant capability for use in the multi-objective optimization of costly functions for the application of excavator structural stress analysis.

A multi-objective genetic algorithm has been used to obtain the Pareto front for the pressure load at the bucket, arm, and boom cylinder. In addition, TOPSIS was applied to determine the best parameter combination among the candidates for the Pareto front. The comparison test results showed that the stress test experiment and the prediction results obtained by using the TOPSIS parameter combination in the FEA model are very similar. Disparities between the observed and predicted (simulated) behaviors are minimized by the proposed multi-objective optimization strategy, despite the measurement errors caused by stress concentrations and sensor locations, as well as modeling errors created by the welded components and local structures. In conclusion, the proposed GPR surrogate model can predict any complicated FEA model, while reducing computational time, and the proposed approach can also investigate the mechanical behavior of the excavator under more complex operating situations; furthermore, the study’s findings provide specific guiding implications for the optimization of the excavator’s main components.

## Figures and Tables

**Table 1:** Material properties of SWS490A steel.

Properties	Value
Yield strength	315 MPa
Poison ratio	0.3
Density	7800 kg/m^3^
Young modulus	210 GPa

**Table 2:** Position setup.

Location	Case-1		Case-2		Case-3	
	Y-Axis [m]	Z-Axis [m]	Y-Axis [m]	Z-Axis [m]	Y-Axis [m]	Z-Axis [m]
(A)	0	0	0	0	0	0
(B)	2.937	0.626	2.917	−0.714	2.917	−0.714
(G)	4.116	−0.450	4.106	−1.780	4.106	−1.780
(J)	4.946	−1.076	4.277	−2.806	4.277	−2.806

**Table 3:** Mesh dense setting.

Parameters	Meshing Size					
	Extra Coarse	Coarse	Normal	Fine	Finer	Extra Fine
Minimum element size [m]	0.367	0.19	0.122	0.068	0.0272	0.0102
Maximum element growth rate	1.85	1.6	1.5	1.45	1.4	1.35
Curvature factor	0.9	0.7	0.6	0.5	0.4	0.3
Resolution of narrow regions	0.2	0.4	0.5	0.6	0.7	0.85

**Table 4:** List of parameters and their ranges.

Parameters	Case-1		Case-2		Case-3	
	lb	ub	lb	ub	lb	ub
Bucket cylinder stroke [mm]	116	126	366	376	264	274
Arm cylinder stroke [mm]	205	215	30	40	479	489
Boom cylinder stroke [mm]	462	472	276	286	597	607
Force angle [deg.]	−15	−5	−10	0	−115	−105

**Table 5:** GPR model verification results summary.

Cases	Responses (Pressure Load)	RMSE [Bar]			
		GBR	SVM [39]	NN [40]	Polynomial
Case-1	at bucket cylinder	0.3638	2.2480	0.4755	9.7452
	at arm cylinder	0.2943	1.2556	2.4571	14.5838
	at boom cylinder	0.1730	2.5538	3.6035	11.9255
Case-2	at bucket cylinder	0.1573	1.0532	1.6700	4.7654
	at arm cylinder	0.2527	1.5985	4.6459	9.5227
	at boom cylinder	0.4518	3.0379	4.1444	5.6509
Case-3	at bucket cylinder	0.9882	1.4911	3.1176	8.7245
	at arm cylinder	0.4790	2.1239	2.2345	9.8825
	at boom cylinder	1.0945	3.0055	4.7070	12.9372

**Table 6:** TOPSIS optimal parameter results.

Parameters	Case-1	Case-2
Bucket cylinder rod stroke [mm]	123.58	375.98
Arm cylinder rod stroke [mm]	214.21	39.86
Boom cylinder rod stroke [mm]	468.20	277.64
Force angle [deg]	−11.37	−8.17

**Table 7:** TOPSIS results for each case.

Cases	Cylinders	Pressure Load [Bar]			Error (Initial Setup) [%]	Error (Optimal Configuration) [%]
		Exp.	Sim. (Initial)	Sim. (Optimal)		
Case-1	Bucket	116.89	119.45	117.04	2.19	0.128
	Arm	193.46	184.52	189.35	4.62	2.125
	Boom	73.35	57.63	67.79	21.43	7.580
Case-2	Bucket	195.04	181.47	192.62	6.96	1.241
	Arm	207.54	223.70	201.28	7.79	3.016
	Boom	216.66	188.47	206.81	12.81	4.546
Case-3	Bucket	53.88	74.68	56.89	38.60	5.586
	Arm	121.59	133.20	121.08	9.55	0.419
	Boom	192.84	210.75	194.04	9.29	0.622

## Data Availability

Dataset available on request from the authors.
